# Serum Zinc Level in Children With Simple Febrile Convulsions

**DOI:** 10.5812/ircmj.4485

**Published:** 2013-07-05

**Authors:** Mehri Taherya, Tahereh Ziaei Kajbaf, Nasrin Janahmadi, Reza Azizi Malamiri, Maedeh Beladi Musavi

**Affiliations:** 1 Department of Paediatrics, Abuzar Children Medical Center, Ahvaz Jundishapur University of Medical Sciences, Ahvaz, IR Iran; 2 Department of Paediatric Neurology, Golestan Medical, Educational, and Research Center, Ahvaz Jundishapur University of Medical Sciences, Ahvaz, IR Iran

**Keywords:** Febrile Convulsion, Zinc, Serum, Children

*Dear Editor,*

Febrile convulsions are the most common convulsive seizures in infants and preschool children (1, 2). Such events, have great impact on the parents and are a leading cause of parental anxiety and family disruption (3). Pathophysiology of febrile convulsions has not been known.2 Genetics and environment have been implicated in their generation (4). Trace elements have been hypothesised to involve in the pathophysiology of febrile convulsions. Recent studies have shown lower post ictal serum zinc levels in children with febrile convulsions (5). Considering the importance of febrile convulsions in our province (south west of Iran) and the theorised implication of hypozincemia in their occurrence, we carried out a case-control study to determine post ictal serum zinc level in a group of children with simple febrile convulsions comparing by those of a control group of febrile children with the same age.

**Figure 1.**
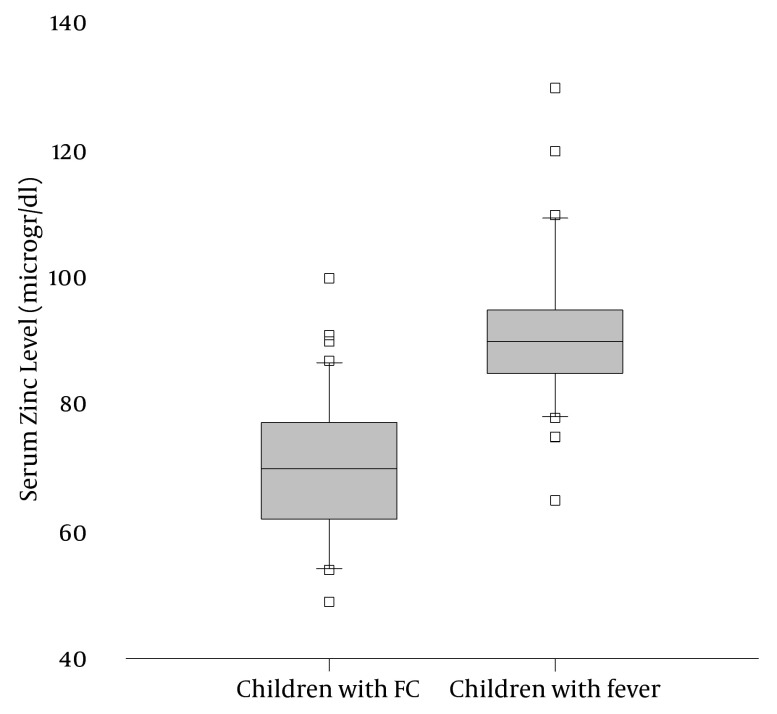
Serum Zinc Level Was Significantly Lower in Children With Febrile Convulsion (40) Than Children With Fever (40) (P < 0.001)

To be enrolled children had to be between 6 months and 5 years old and had to meet the diagnostic criteria of simple febrile convulsions according to the International League against Epilepsy definition (6). Excluded were those who received zinc supplements. The control group was comprised of children less than 5 years who were seen in the outpatient clinic or emergency department because of a febrile illness other than central nervous system infection. Informed consent was obtained from the parents of all children before enrolment into the study. Blood samples (2 ml) were drawn by venipuncture from the antecubital vein using a disposal plastic syringe with a 23 gauge needle in the morning between 8:00 and 10:00 am. All children with febrile convulsions were at least 24 hours seizure-free from the sampling time. Blood samples were centrifuged at 1500 RPM for 10 min, and the serum was stored at -86ºC until the time of analysis. Serum zinc concentration was measured by the graphite furnace flame atomic absorption spectrophotometry (AAS; SpectrAA 220, GTA 110, Varian, Australia) technique. Serum zinc concentration was reported as microgr/dl. Statistical analysis was performed using SPSS version 16.0 statistical software (SPSS, Inc, Chicago, IL). Categorical variables were analysed using the χ2 test. Continuous variables were analysed using independent sample t-test, or Mann-Whitney Rank Sum test when appropriate. A P < 0.05 was considered significant. Eighty children (40 children in each group) aged 9 months to 5 years were enrolled into this study. In both groups the majority of participants were aged between 2 and 4 years. Children in both groups were not significantly different for age, sex, and nutrition (P > 0.05). In both groups the most frequent cause of fever was otitis media and other upper respiratory tract infections. No significant difference was found between two groups in terms of aetiology of fever. In the patients’ group, serum zinc level (median; 70 microgr/dl) was significantly lower than the control group (median; 90 microgr/dl) (P < 0.001) (Figure 1).

In both groups, no relation was found between serum zinc level and age. In both groups, no relation was found between serum zinc level and aetiology of fever. Our results indicate that low or reduced postictal serum zinc level in children with febrile convulsion is a reproducible finding in different geographical regions of the world. Moreover, no relation was found between aetiology of fever and serum zinc level. Recently, hypozincemia and reduced levels of CSF zinc have been reported in children who had febrile convulsions (5). However, exact role of the zinc in the pathogenesis of febrile convulsions is still unknown. Previous studies have been proposed a theory about induction of febrile convulsions with a reduced level of serum zinc level. Based on this theory, hypozincemia activates the NMDA receptor, one of the glutamate families of receptors, which may play an important role in inducting epileptic discharge.5 Moreover, zinc has a regulatory effect on glutamic acid decarboxylase and this enzyme is the core in the synthesis of GABA. Decreased function of the mentioned enzyme has been hypothesized in hypozincemia. Therefore, hypozincemia could lead to reduced levels of GABA in the brain and finally seizure threshold could be reduced in conditions with hypozincemia.

The major limit of this study was that we only measured serum zinc level during a febrile illness; therefore we could not compare the serum zinc level of our participants during illness and healthy states. Few studies with the same methods were conducted in different countries to measure the serum zinc level in children with febrile convulsions. In all of these studies, results were shown that children with simple febrile convulsions had serum zinc levels lower than that of febrile children with the same age (7, 8). In conclusion, a considerable body of evidence now exists that shows hypozincemia in children with febrile convulsions during an episode of seizure. We think it is valuable to design large prospective trials to assess the serum zinc level in children at risk for the occurrence of febrile convulsions during healthy states and before a seizure occurrence.
